# Sex and age differences in mechanical, thermal and wetness perception across skin sites at risk of pressure ulcers: A quantitative sensory testing study

**DOI:** 10.1113/EP093190

**Published:** 2026-03-15

**Authors:** Miriam Martini, Arianna Ortis, Davide Filingeri, Peter R. Worsley, Evelin Makuc, Manuela Deodato, Luigi Murena, Paolo Manganotti, Alex Buoite Stella

**Affiliations:** ^1^ School of Physiotherapy, Department of Medicine, Surgery and Health Sciences University of Trieste Trieste Italy; ^2^ PhD program in Personalized Medicine and Innovative Therapies, Department of Medicine, Surgery and Health Sciences University of Trieste Trieste Italy; ^3^ THERMOSENSELAB, Skin Sensing Research Group, School of Health Sciences The University of Southampton Southampton UK; ^4^ Plastic and Reconstructive Surgery Unit, Department of Medicine, Surgery and Health Sciences, Cattinara University Hospital – ASUGI University of Trieste Trieste Italy; ^5^ Orthopedics and Traumatology unit, Department of Medicine, Surgery and Health Sciences University of Trieste Trieste Italy; ^6^ Clinical Unit of Neurology, Department of Medicine, Surgery and Health Sciences, Cattinara University Hospital – ASUGI University of Trieste Trieste Italy

**Keywords:** ageing, hygrosensation, pain, pressure injury, sex difference, thermal sensation

## Abstract

Pressure ulcers can be common in the elderly, and loss of sensation can play a fundamental role in the development of these injuries. The aim of this study was to assess the effects of age and sex on non‐noxious thermal and wetness perception (via magnitude estimation), alongside pressure‐ and thermal‐pain thresholds, at the heel, sternum and sacrum, which are skin sites typically at risk for pressure ulcers. A cross‐sectional study was performed on 34 younger adults [15 females, 22.1 (1.7) years old] and 17 older adults [eight females, 55.5 (5.3) years old]. We found that: (1) pressure pain thresholds (*F*_2,24_ = 16.60, *P *< 0.001) and heat pain thresholds (*F*_2,24_ = 4.23, *P* = 0.027) differed in relationship to age, but only at the heel and sacrum, principally with higher thresholds in the older adults. Furthermore, when collapsed by skin site, we found that females had lower pressure pain thresholds (−157 kPa; *P* = 0.011) and heat pain thresholds (−1.54°C; *P* = 0.008) than males. Considering hygrosensation, it was typically higher in the young adults, and the heel was the skin site least sensitive to both thermal and hygrosensation. The results of this study indicate that sensory alterations could be present in skin sites at risk of pressure ulcers; in particular, the heel presented less intensity of sensations to painful and non‐noxious stimuli. As such, according to these findings, male sex and age can result in reduced intensities of sensations, which might predispose to a higher risk of pressure ulcers, especially on the heel.

## Abstract (highlights)

- **What is the central question of this study?**
  What are the sex‐ and age‐related differences in mechanical, thermal and wetness perception in skin areas at risk of pressure ulcers?
- **What is the main finding and its importance?**
  The heel represents a poorly sensitive area. Male sex and age are associated with generally lower intensity of sensations, therefore predisposing to a higher risk of development of pressure ulcers.

## 1 INTRODUCTION

Pressure ulcers are defined as localized injuries to the skin and/or underlying tissues, which generally occur over bony prominences as a result of sustained pressure or pressure in combination with shear forces (Kottner et al., 2009). Soft tissue tolerance to these stimuli can be influenced by several factors, such as microclimate (i.e., temperature, humidity and airflow near the skin), nutrition, skin perfusion, soft tissue conditions and other comorbidities (Edsberg et al., 2016). Pressure ulcers represent a healthcare problem throughout the world because they have a strong impact on the quality of life of individuals who suffer from them (Demarré et al., 2015). The reported prevalence of pressure ulcers varies from 14% to 22% in intensive care units, whereas in the general hospital setting the prevalence is ∼9% (De Oliveira et al., 2019). Previous research investigating the prevalence of pressure ulcers was conducted mainly in the elderly, and therefore there is a lack of data regarding the prevalence of pressure ulcers in younger people; however, it was suggested that up to the age of 50 years, males might present a higher prevalence of pressure ulcers owing to the greater prevalence of traumatic spinal cord injuries that cause immobility (Mervis & Phillips, 2019). In contrast, no differences were reported between sexes in older adults, between 70 and 80 years of age, with an overall prevalence of pressure ulcers of ∼29% (Mervis & Phillips, 2019).

Various risk factors for the development of pressure ulcers have been identified and are grouped into extrinsic factors (e.g., pressure, friction and shear forces) and intrinsic factors (e.g., immobility, malnutrition and reduced skin perfusion) (Chou et al., 2013). Amongst the extrinsic factors, microclimate humidity and the associated build‐up of moisture at the skin interface play an important role (Russell, 1998). Indeed, repeated and prolonged exposure to sweat or other body fluids, such as faeces or urine, could promote skin maceration, reduce skin tissue integrity and, ultimately, predispose the skin to superficial ulceration (Beeckman et al., 2014). Amongst the intrinsic factors, sensory loss also seems to play a fundamental role, because it could reduce the capacity of an individual to perceive the discomfort or pain resulting from prolonged pressure and trigger protective behaviours (e.g., postural changes and skin drying) (Berman, 2018; Russell, 1998). As such, skin sensitivity is essential to detect extrinsic factors that can cause damage and could be altered as part of the intrinsic factors associated with development of pressure ulcers (Mervis & Phillips, 2019).

In the pathogenesis of pressure ulcers, the role of pressure, friction and shear forces is well known, and their conscious perception triggers a feedback response leading to postural changes and pressure release. However, when pressure or pain sensitivity is altered, for example as a result of ageing or sensory loss (Mervis & Phillips, 2019), this response could be absent or poor, resulting in increased risk of ischaemic damage to the superficial skin tissue (Case et al., 2021; Handler & Ginty, 2021; Mervis & Phillips, 2019). Besides pressure‐related sensations, the perception of temperature also represents a fundamental sensory input that allows us to avoid potentially harmful thermal conditions (McKemy, 2005; McKemy et al., 2002; Morin & Bushnell, 1998; Patapoutian et al., 2003; Rolls et al., 2008; Wetsel, 2011). Furthermore, in the literature, there is evidence regarding the variability of skin sensitivity between different body areas. In particular, temperature sensitivity is not uniform throughout the body, varying significantly depending on the region of the skin being stimulated (Gerrett et al., 2014; Valenza et al., 2019). However, to the best of our knowledge, there are no data regarding skin sensitivity regional differences in skin sites that are typically at risk of developing pressure ulcers.

Furthermore, females appear to be more thermosensitive than males (Gerrett et al., 2014). In preventing the development of pressure ulcers, the perception of humidity (i.e., hygrosensation) could also play an important role, because it contributes to the awareness of one's own thermal/wetness state and the surrounding environment (Filingeri et al., 2015; Valenza et al., 2019). Temperature perception plays a key role in the perception of wetness. Humans are more likely to perceive cold humidity rather than stimuli of equal humidity but at higher temperatures. Females appear to be 14%–17% more sensitive to cold humidity, and this agrees with the fact that females are also 8%–14% more sensitive to cold (Buoite Stella et al., 2022; Filingeri, 2014; Filingeri & Havenith, 2018). In addition, morphological changes related to ageing, such as dehydration, altered skin elasticity, reduction in peripheral blood flow and decrease in the number of myelinated afferent fibres and skin receptors, can lead to alterations in tissue sensitivity (Boismal et al., 2020; Bonté et al., 2019; Bowden & McNulty, 2013a, 2013b; Carmeli et al., 2003; Wildgoose et al., 2021). It is believed that skin sensitivity and the perception of pain decrease with advancing age in healthy subjects, thus it could increase the risk of developing pressure ulcers (Bonté et al., 2019; Bowden & McNulty, 2013a; Cole et al., 2010; El Tumi et al., 2017; Gibson et al., 1991; Kemp et al., 2014; Larivière et al., 2007; Lautenbacher et al., 2005; Pickering et al., 2002).

Despite several studies having investigated alterations in skin sensitivity in different conditions and populations (Cole et al., 2010; Gibson et al., 1991; Larivière et al., 2007; Lautenbacher et al., 2005; Pickering et al., 2002), most of these studies have investigated body areas that are not typically subject to pressure ulcers; therefore, there is a lack of data on sex and age differences in mechanical, thermal and wetness perception across skin sites at risk of pressure ulcers, which could inform individualized risk profiles for pressure injury that account for variations in skin sensitivity. It might be hypothesized that ageing reduces skin perception to these stimuli and that females present lower thresholds. As such, the aim of this study was to assess the individual and interactive effects of sex and age in non‐noxious thermal and hygrosensation, in addition to mechanical, heat and cold pain thresholds, across skin sites typically at risk of developing pressure ulcers, such as the sternum, the sacrum and the heel.

## 2 MATERIALS AND METHODS

### 2.1 Participants

**FIGURE 1:**
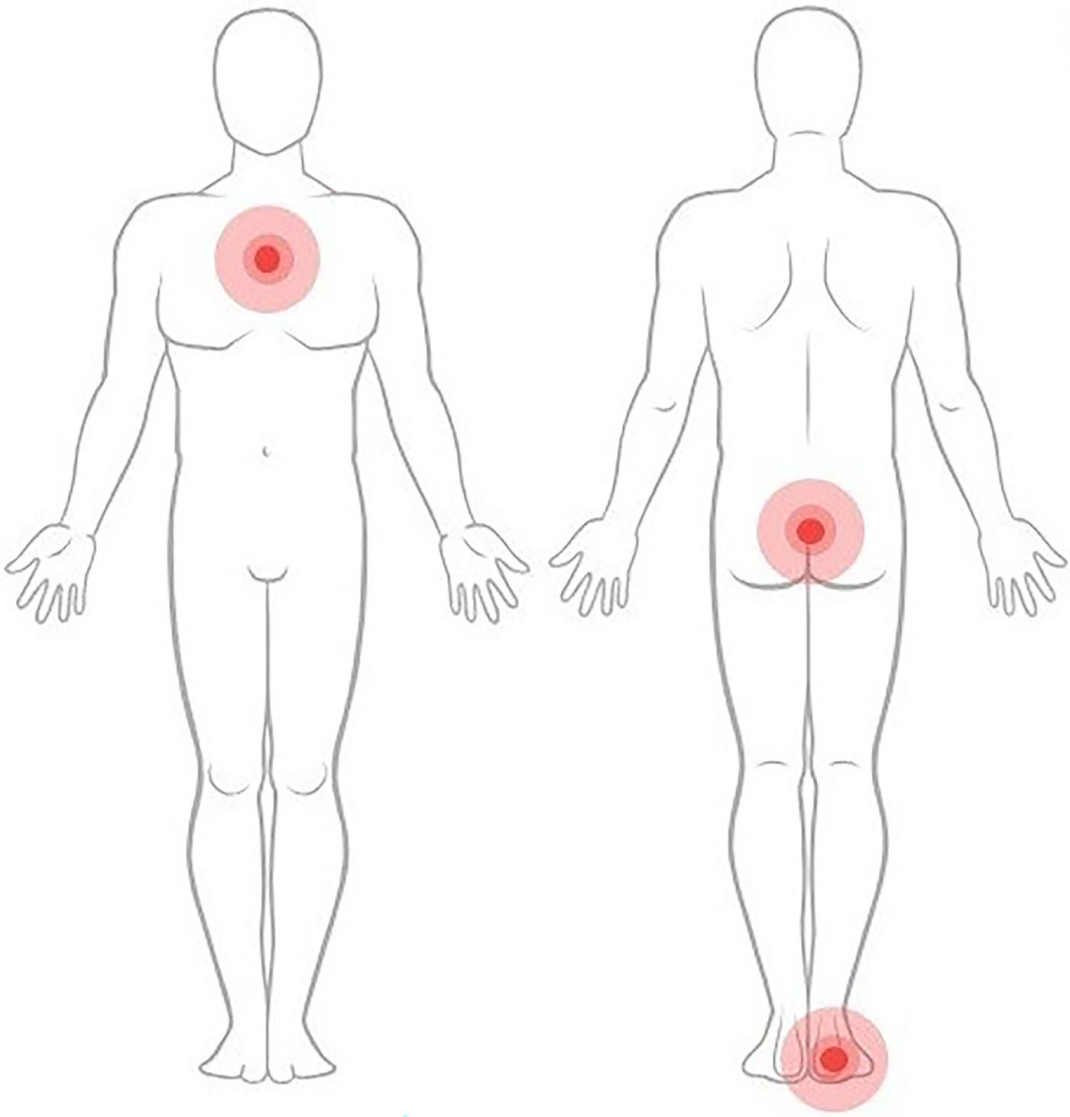
Representation of the three skin sites evaluated in this study: the sternum, sacrum and dominant heel.

A cross‐sectional study was conducted from June 2023 to September 2023, in a University Hospital setting. Based on previous works, and considering an effect size (*f*  = 0.25), power of 0.80 and α of 0.05, a sample size of 12 was considered sufficient for one between‐participant variable (i.e., group) and two within‐participant variables (i.e., stimulation and intensity) (Zhi et al., 2024). Considering that in the present study both age and sex groups were compared, we aimed to recruit ≥12 subjects for each category (i.e., 12 males, 12 females, 12 young and 12 old adults). To take part in the study, participants had to be >18 years of age. Exclusion criteria were the presence of peripheral neuropathies, suffering from alterations in sensation or vascularization, having undergone surgery at the sites assessed, and a history of previous pressure ulcers at the assessed sites. Furthermore, only non‐smoking individuals were included in this study to ensure better homogeneity of the sample. A neurologist (P.M.) evaluated the medical history of the participants, and individuals were also excluded if taking medications affecting skin sensitivity or if other comorbidities were reported. Considering the inclusion and exclusion criteria, 51 individuals were enrolled and were grouped based on sex and age as follows: (1) old adults [i.e., adults >50 years, up to 70 years; *n* = 17, eight females, 55.5 (5.3) years]; and (2) young adults [i.e., adults between 18 and 30 years of age; *n* = 34, 15 females, 22.1 (1.7) years] (Table 1). Age groups were partly based on previous literature to highlight differences between young adults and middle‐aged/older adults (Zhi et al., 2024). All the included participants were invited to a single testing session to evaluate mechanical, thermal and wetness perception on the sternum, sacrum and heel (on their dominant side) (Figure 1).

**TABLE 1:** Demographic and anthropometric characteristics of the included sample represented as age and sex subgroups.

	Young adults (*n* = 34)		Old adults (*n* = 17)	
Characteristic	M (*n* = 19)	F (*n* = 15)	M (*n* = 9)	F (*n* = 8)
Age, years	22.10 ± 1.70	22.70 ± 1.20	57.20 ± 6.40	53.60 ± 2.60
BM, kg	73.10 ± 10.90	64.40 ± 11.30	82.50 ± 8.60	72.30 ± 13.00
Height, m	1.77 ± 0.84	1.70 ± 0.78	1.81 ± 0.42	1.67 ± 4.30
BMI, kg/m^2^	23.6 ± 1.5	21.5 ± 3.7	25.2 ± 2.7	25.9 ± 3.6
Right handedness, *n* (%)	17 (89)	14 (93)	9 (100)	7 (88)

*Note*: Data are reported as means and SDs except where indicated otherwise.

Abbreviations: BM, body mass; BMI, body mass index; F, female; M, male; TOT, total subjects in the group.

### 2.2 Experimental design

Three trials were performed on each area evaluated, and the average of the three values ​​was considered. The three skin sites were evaluated in a randomized order, and based on previous literature, 1 min of rest was allowed between each measure to avoid possible peripheral sensitization that could occur with stimulations too close (Deodato, Granato, Ceschin et al., 2022; Filingeri et al., 2014, 2018). All the assessments were performed in the same room, kept between 24°C and 26°C and between 20% and 40% relative humidity, at the same time of the day for all subjects (between 14:00 and 17:00 h). Participants were positioned on a physiotherapy bed, in a comfortable position, lying on their back or chest according to the tested skin site. Three female participants within the old adult group reported being in a postmenopausal phase, whereas the other female participants were tested during the self‐reported follicular phase of their menstrual cycle for standardization, and they reported having a regular menstrual cycle and were not consuming contraceptive pills or other drugs. All the participants were instructed to avoid exercise or thermal therapies (e.g., sauna or hot/cold baths) in the previous 24 h and not to use any cream or gel on the skin during the day of the assessment. They were asked to refrain from consuming alcohol in the 24 h preceding testing, and from smoking and consuming caffeine or food in the 3 h preceding testing.

The study was approved by the institutional review board (134/2023), and it was performed in accordance with the *Declaration of Helsinki*. Informed consent was obtained from all subjects, and the privacy rights of all subjects were protected.

#### 2.2.1 Pressure pain threshold

To investigate pressure pain threshold (PPT) in response to mechanical stimuli, PPT was assessed with an algometer (Somedic Sales, Hörby, Sweden) on the three skin sites evaluated. The algometer was placed perpendicularly, with the probe (circular area of 1 cm^2^) on the bony protuberance in the investigated area, according to standard procedures, and the pressure was increased at a rate of 30 kPa/s (Deodato, Granato, Borgino et al., 2022, 2022; Linde et al., 2018). In detail, a visual feedback provides the assessor with a standardization of the increasing force (pressure) applied to the specific area. Participants were instructed to press a specific button as soon as the mechanical stimuli were perceived as painful. Before starting the procedure, the first trial was applied on the dominant hand of each participant to familiarize them with the procedures. The pressure value was saved automatically in the dedicated software on the computer connected to the algometer. The average of the values was calculated for each point and considered in the final analysis.

#### 2.2.2 Heat pain threshold and cold pain threshold

To evaluate pain thresholds to heat and cold, the thermal sensitivity tester was used by applying the circular probe (diameter 13 mm) on the reference skin sites. The thermal probe uses a Peltier semiconductor heat pump to provide temperature stimuli above and below ambient temperature. The temperature range of the thermode extends from 0°C to 50°C, and a digital display indicates the temperature at the probe tip with a resolution of 0.1°C (Buoite Stella et al., 2022; Valenza et al., 2019; Wildgoose et al., 2021). In this study, the starting temperature was set at 32°C for all three skin sites. Limit temperatures were chosen to avoid possible injuries to the subjects evaluated, that is, 48°C to evaluate heat pain threshold (HPT) and 5°C to evaluate cold pain threshold (CPT). If a subject did not report pain or sensation before the limit temperature, the limit temperature was then reported as >48°C or <5°C.

Once the instrumentation was set up, it was explained to the subject that the probe would be placed on the evaluation site and that the temperature would vary by 1°C/s, reaching the selected temperature by heating or cooling. The participants were instructed to press a button when the thermal stimulus was perceived as painful to interrupt the evaluation and automatically save the data. The temperature read on the display was recorded and averaged for the final analysis.

#### 2.2.3 Non‐painful thermal and hygrosensation

To evaluate non‐noxious thermal perception and hygrosensation, the thermal sensitivity tester was used according to previous protocols from our group (Buoite Stella et al., 2022; Valenza et al., 2019; Wildgoose et al., 2021). For this evaluation, the probe was wrapped in a gauze soaked in 0.8 mL of water, and three different temperatures were set on the machine to result in neutral wet (32°C), warm wet (37°C) and cold wet (27°C) stimuli, according to previous literature (Buoite Stella et al., 2022; Valenza et al., 2019; Wildgoose et al., 2021). The participants were informed that the probe would be placed on the three skin sites for 3 s, at the end of which they had to complete two visual analog scales (VAS) based on previous research (Buoite Stella et al., 2022; Valenza et al., 2019; Wildgoose et al., 2021). The first VAS (score 0–200) was related to thermal sensation, where 0 represented cold, 100 neutral temperature, and 200 hot. Therefore, the participants rated their thermal sensation to neutral, warm and cold wet stimuli (NT, WT and CT, respectively). The second VAS (score 0–100) was related to wetness perception, in which 0 represented dry and 100 represented completely wet. Therefore, the participants rated their hygrosensation to neutral, warm and cold wet stimuli (NH, WH and CH, respectively). The three temperatures were evaluated in a randomized order (the assessor drew a coloured card from a box), and the collected scores were averaged for the final analysis.

### 2.3 Statistical analysis

The statistical analysis was performed with SPSS v.23 (IBM Inc.). The Shapiro–Wilk test for normality of distribution was performed. Data are reported as means and SDs. A mixed‐factors ANOVA was performed to test within‐subject factors (three skin sites/areas: heel, sternum and sacrum) and between‐subject factors (two sexes: female and male; and two ages: younger and older adults). In the event of significant main effects, a Bonferroni *post hoc* comparison was performed. The effect size was reported as partial eta‐squared (η^2^_p_). Significance was set at *P *< 0.05. Graphical representation was performed with the software GraphPad Prism v.8.4.1.

## 3 RESULTS

Characteristics of participants are reported in Table 1. The results from the quantitative sensory testing are reported in Table 2 for each subgroup and skin site/area.

**TABLE 2:** Quantitative sensory assessment of the select skin sites/area (heel, sacrum and sternum) represented as age and sex subgroups.

Variable	Area	Young adults (*n* = 34)		Old adults (*n* = 17)	
		M (*n* = 19)	F (*n* = 15)	M (*n* = 9)	F (*n* = 8)
PPT^*^, kPa	Heel^†^	1318.30 ± 278.40	1216.50 ± 259.30	1088.30 ± 133.90	944.80 ± 15.00
	Sacrum^†^	785.70 ± 286.60	787.00 ± 213.40	1033.20 ± 207.40	822.50 ± 153.20
	Sternum	656.20 ± 327.20	488.30 ± 198.50	684.50 ± 171.50	364.30 ± 88.00
HPT^*^, °C	Heel^†^	44.70 ± 2.20	43.20 ± 1.70	47.00 ± 0.60	45.40 ± 1.90
	Sacrum^†^	39.00 ± 2.40	38.20 ± 1.50	41.20 ± 2.90	38.80 ± 1.00
	Sternum	39.40 ± 3.40	39.60 ± 2.40	41.60 ± 2.70	38.30 ± 1.80
CPT, °C	Heel	5.60 ± 1.90	5.00 ± 0.00	<5.00	<5.00
	Sacrum	5.80 ± 3.00	5.10 ± 0.50	<5.00	<5.00
	Sternum	5.40 ± 1.60	5.20 ± 0.60	<5.00	<5.00
WT, score	Heel	122.70 ± 26.80	122.20 ± 37.60	105.00 ± 20.80	105.80 ± 9.90
	Sacrum^a^	118.70 ± 30.90	140.40 ± 32.00	128.10 ± 32.80	123.80 ± 20.80
	Sternum^a^	121.50 ± 17.30	130.40 ± 21.90	124.70 ± 18.50	126.60 ± 11.40
WH^#^, score	Heel	20.20 ± 16.70	29.40 ± 30.10	20.20 ± 16.70	17.20 ± 15.00
	Sacrum^b^	33.00 ± 18.70	38.30 ± 19.80	14.70 ± 15.40	17.20 ± 12.10
	Sternum	14.90 ± 9.50	14.00 ± 11.30	12.20 ± 9.70	12.20 ± 8.80
NT^#^, score	Heel	95.00 ± 9.90	100.90 ± 24.40	99.40 ± 6.80	102.50 ± 7.60
	Sacrum	87.40 ± 18.10	96.20 ± 15.10	98.10 ± 3.00	101.30 ± 16.40
	Sternum	91.10 ± 22.90	96.80 ± 13.50	102.50 ± 13.90	104.70 ± 22.20
NH^#^, score	Heel	28.90 ± 20.80	32.10 ± 29.70	15.80 ± 17.60	11.60 ± 10.90
	Sacrum	32.70 ± 18.90	32.60 ± 23.20	19.20 ± 17.00	14.40 ± 13.50
	Sternum	37.80 ± 22.50	35.90 ± 28.60	15.00 ± 16.30	11.90 ± 8.00
CT^#^, score	Heel	55.90 ± 29.00	48.90 ± 40.30	90.00 ± 17.90	69.70 ± 32.20
	Sacrum	53.80 ± 21.60	50.20 ± 22.60	58.30 ± 20.80	75.30 ± 34.00
	Sternum	59.20 ± 20.00	49.90 ± 27.20	53.60 ± 25.90	74.70 ± 18.70
CH^#^, score	Heel	53.20 ± 26.60	58.90 ± 31.30	27.20 ± 24.30	37.50 ± 22.50
	Sacrum^a^	66.40 ± 16.10	66.10 ± 18.40	46.40 ± 32.10	47.80 ± 25.70
	Sternum	51.90 ± 23.50	64.80 ± 24.50	36.10 ± 28.50	42.50 ± 12.50

*Note*: Data are reported as means and SDs except where indicated otherwise. Note that for thermal sensation, the scores should be interpreted according to the visual analog scale (0, cold; 100, neutral; 200, hot), and for the hygrosensation the visual analog scale (0, dry; 100, completely wet).

Abbreviations: CPT, cold pain threshold; CT, cold temperature; CW, cold hygrosensation; HPT, heat pain threshold; NH, neutral hygrosensation; NT, neutral temperature; PPT, pressure pain threshold; WH, warm hygrosensation; WT, warm temperature.

Significant differences (*P *< 0.05) in the mixed‐factors ANOVA are highlighted as follows:

* sex effect (independent from age and skin site);

^#^ age effect (independent from sex and skin site);

^†^ age × skin site/area interaction;

^a^ area effect, greater than the heel (independent from sex and age); and

^b^ area effect, greater than the sternum (independent from sex and age).

### 3.1 PPT

**FIGURE 2:**
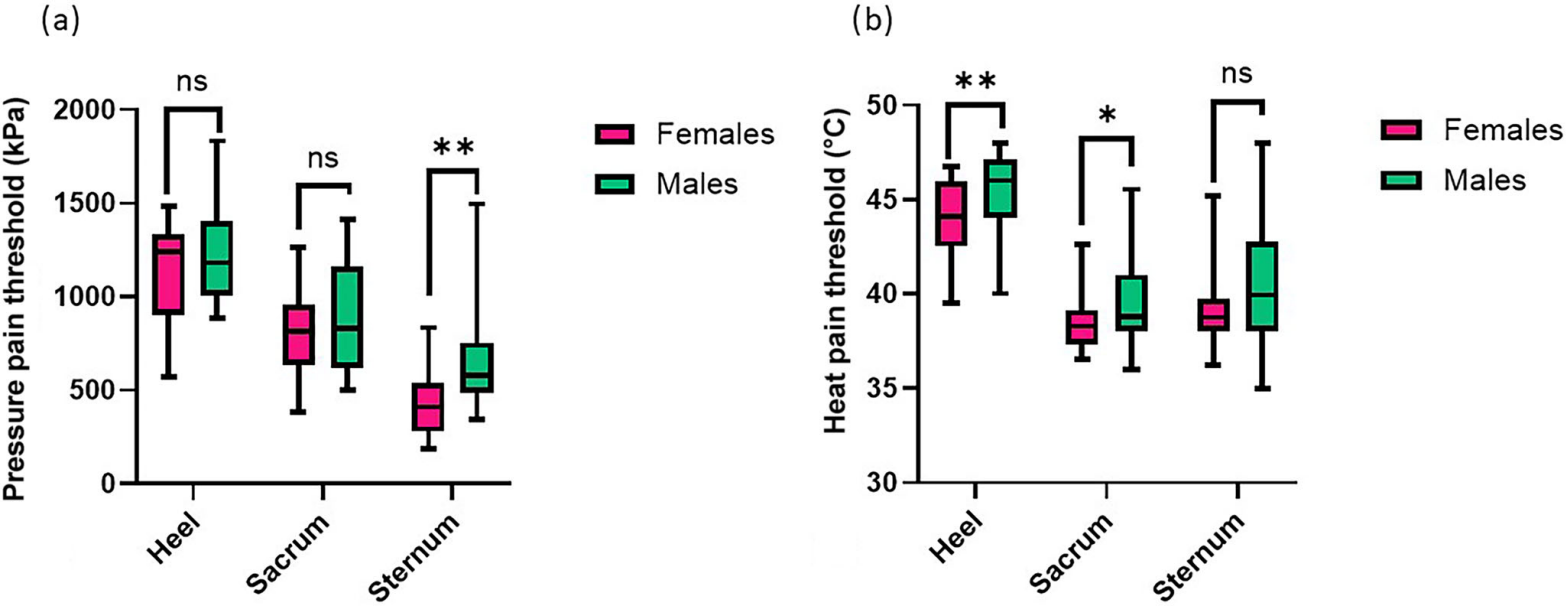
Boxplots comparing the three skin sites between females (magenta, *n* = 23) and males (green, *n* = 28), regarding the pressure pain threshold (a) and the heat pain threshold (b). A mixed‐factors ANOVA was performed, and significance is reported for the sex effect within the single skin site as follows: ns, not significant; *P < 0.05; and **P < 0.01.

PPT were assessed in the different skin sites and compared based on age and sex. No interaction effects for area × sex × age, area × sex or sex × age were found for PPT, but we found a significant area × age interaction effect (*F*_2,24_ = 16.60, *P *< 0.001, η^2^_p_ = 0.26). Specifically, PPT were found to be greater in younger compared with older adults at the heel (mean difference: 250.90 kPa, 95% CI: 108.84–392.95; *P* = 0.001), whereas PPT were greater in older than younger adults at the sacrum (mean difference: 141.49 kPa, 95% CI: 0.22–283.22; *P* = 0.050). No statistically significant differences were found between the two age groups at the sternum (*P* = 0.510; Figure 2a). A statistically significant main effect of sex was also found (*F*_1,47_ = 7.01, *P* = 0.011, η^2^_p_ = 0.13), such that, irrespective of age and skin site, males presented greater PPT than females, with this mean difference being 157.11 kPa (95% CI: 37.79–276.42; *P* = 0.011; Figure 2b). Taken together, these findings indicate that PPT is modulated by an interaction between age and skin site such that for the tested sites, older adults exhibit a ∼20% lower PPT at the heel and a ∼18% higher PPT at the sacrum than the younger cohort. Moreover, males were characterized by ∼20% higher PPT than females.

### 3.2 HPT and CPT

Thermal pain thresholds were assessed in the different skin sites and compared based on age and sex groups. No significance was highlighted for CPT in all interactions and single effects. No interaction effects for area × sex × age and area × sex or sex × age were reported for HPT but a significant area × age effect was recorded (*F*_2,24_ = 4.23, *P* = 0.027, η^2^_p_ = 0.83). In particular, independent from sex, it was found to be higher in the old adults than in the young adults group, both on the heel (mean difference: 2.34°C, 95% CI: 1.23–3.45; *P *< 0.001) and the sacrum (mean difference: 1.33°C, 95% CI: 0.70–2.59; *P* = 0.039); in contrast, no significant difference was found on the sternum between old adults and young adults (*P* = 0.660). A significant sex effect was found for HPT (*F*_1,47_ = 7.65, *P* = 0.008, η^2^_p_ = 0.14), whereby males presented higher values than females, with a mean difference of 1.54°C (95% CI: 0.42–2.67; *P* = 0.008; Figure 2). Taken together, these findings indicate that, excluding the sternum, HPT is modulated independently by age and sex, whereby older adults exhibit ∼5% and ∼3% higher HPT to heat pain than younger adults at the heel and sacrum, respectively, and males were characterized by ∼4% higher HPT than females.

### 3.3 Non‐painful thermal and hygrosensation

**FIGURE 3:**
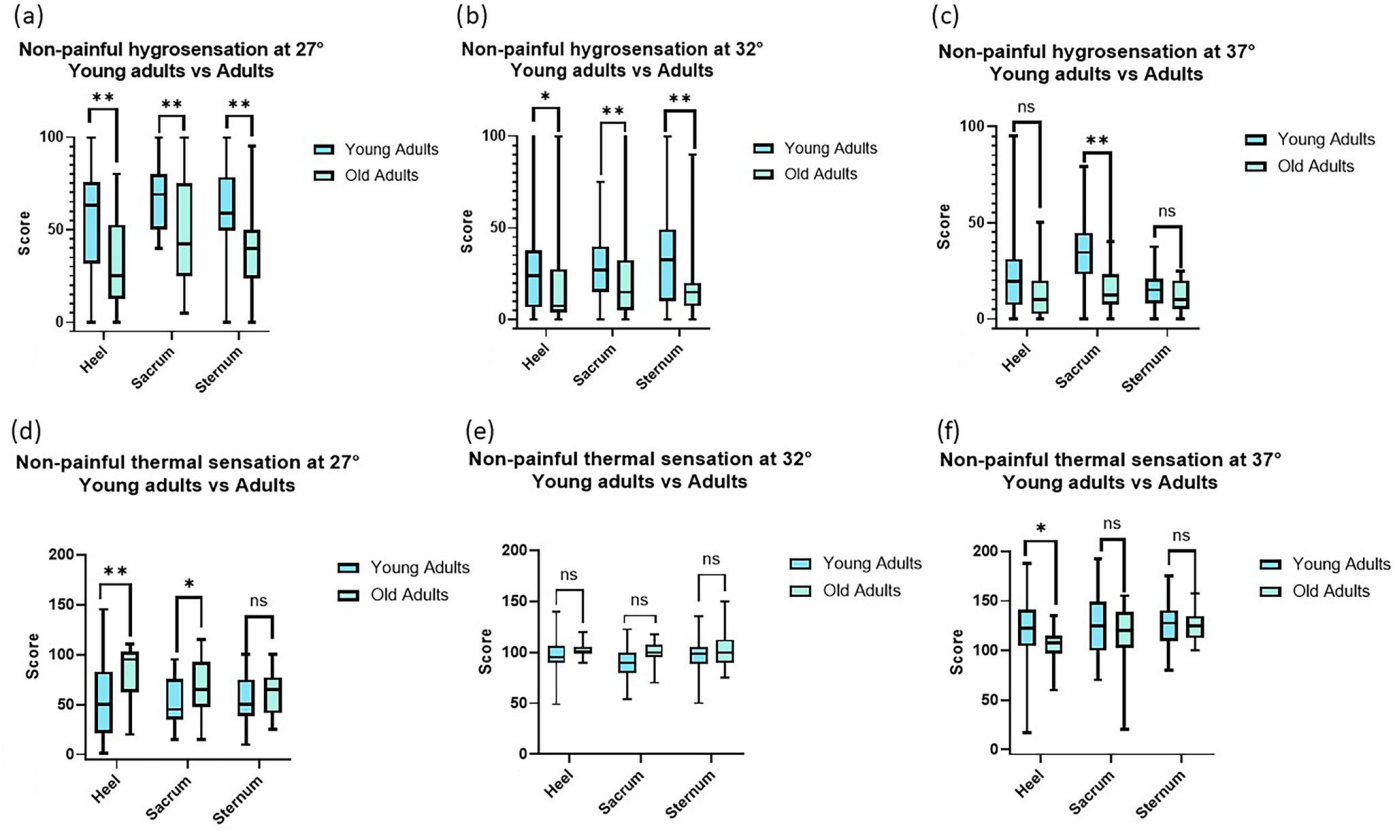
Boxplots comparing the three skin sites between young adults (light blue, *n* = 34) and old adults (green, *n* = 17), regarding: (a) non‐painful hygrosensation at 27°C (CH; 0, dry–100, wet); (b) non‐painful hygrosensation at 32°C (NH; 0, dry–100, wet); (c) non‐painful hygrosensation at 37°C (WH; 0, dry–100, wet); (d) non‐painful thermal sensation at 27°C (CT; 0, cold–100, neutral–200, hot); (e) non‐painful thermal sensation at 32°C (NT; 0, cold–100, neutral–200, hot); and (f) non‐painful thermal sensation at 37°C (WT; 0, cold–100, neutral–200, hot). A mixed‐factors ANOVA was performed, and significance is reported for the age effect within the single skin site as follows: ns, not significant; *P < 0.05; and **P < 0.01.

Thermal and hygrosensation were assessed in the different skin sites and compared based on age and sex groups. No interaction effect for area × sex × age and area × sex or sex × age were highlighted for non‐painful thermal and hygrosenstion, but a significant age effect was reported. In particular, the perception of cold wetness (*F*_1,47_ = 12.83, *P* = 0.001, η^2^_p_ = 0.21) was greater in magnitude in the young adult group than the old adult group by 20.63 mm (95% CI: 9.04–32.20; Figure 3a); the perception of neutral wetness (*F*_1,47_ = 14.15, *P *< 0.001, η^2^_p_ = 0.23) was greater in magnitude in the young adult group than the old adult group by 18.71 mm (95% CI: 8.70–28.72; Figure 3b); the perception of warm wetness (*F*_1,47_ = 10.52, *P* = 0.002, η^2^_p_ = 0.18) was greater in magnitude in the young adult group than the old adult group by 10.95 mm (95% CI: 4.16–17.75; Figure 3c); the thermal perception of cold stimulus (*F*_1,47_ = 7.64, *P* = 0.008, η^2^_p_ = 0.14) was less cold in the old adult group than the young adult group by 17.27 mm (95% CI: 4.70–29.84; Figure 3d); and the thermal perception of neutral stimulus (*F*_1,47_ = 7.16, *P* = 0.010, η^2^_p_ = 0.13) was warmer in the old adult group than the young adult group by 6.82 mm (95% CI: 1.69–11.95; Figure 3e). In contrast, no significant age effect was found for the thermal perception of warm stimulus (*F*_1,47_ = 2.07, *P* = 0.150, η^2^_p_ = 0.04; Figure 3f).

A significant area effect was found for the thermal perception of warm stimulus (*F*_2,94_ = 4.61, *P* = 0.013, η^2^_p_ = 0.08), the perception of warm wetness (*F*_2,94_ = 7.50, *P* = 0.001, η^2^_p_ = 0.13) and the perception of cold wetness (*F*_2,94_ = 5.53, *P* = 0.006, η^2^_p_ = 0.10). In particular, the thermal perception of warm stimulus was warmer in the sacrum compared with the heel (13.80 mm, 95% CI: 0.63–26.97, *P* = 0.037) and warmer in the sternum compared with the heel (11.85 mm, 95% CI: 0.22–23.49, *P* = 0.044); the perception of warm wetness was significantly greater in magnitude in the sacrum than in the sternum by 12.48 mm (95% CI: 5.04–19.91, *P *< 0.001), and the perception of cold wetness was greater in magnitude in the sacrum than the heel with a difference of 12.48 mm (95% CI: 2.33–22.63, *P* = 0.011). Taken together, these findings indicate that hygrosensation is independently modulated by age, and older adults exhibit a ∼44% lower intensity of sensation to wetness.

## 4 DISCUSSION

The aim of this study was to investigate the role of sex and age differences in mechanical, thermal and wetness perception across skin sites at risk of pressure ulcers, namely the sternum, sacrum and heel, using quantitative sensory testing. Our findings provided several new insights into the interaction of sex and age on local perceptual responses to noxious and non‐noxious thermal and mechanical stimuli, in otherwise healthy adults. Indeed, sensitivity to both noxious and non‐noxious stimuli could be involved in the pathophysiology of pressure ulcers, describing possible different mechanisms (Mervis & Phillips, 2019); in this study, age was associated with a decrease in thermal noxious perception (heat pain) and a reduction of cold and wetness non‐noxious perception, whereas mechanical pain (PPT) was influenced by the skin site (older adults being more sensitive to pain on the heel and less sensitive on the sacrum). Sex and skin sites were found primarily to influence the two noxious perception outcomes (higher thresholds in males and at the heel), with limited effects on the non‐noxious thermal and wetness outcomes.

First, we identified that, regardless of age and skin site, female participants presented greater intensity of sensation to heat and pressure pain than males, as evidenced by lower values ​​of the HPT (∼4%) and PPT (∼17%). This result is in line with previous studies, which identified the interaction amongst biological, psychological and sociocultural factors potentially to underlie these sex‐based differences (Bartley & Fillingim, 2013). Sex hormones and their receptors have previously been associated with nociceptive activity, which might also influence the differential activation of the brain during the processing of pain‐related stimuli (Craft, 2007; Smith et al., 2006). Although female sex hormones, such as estradiol and progesterone, seem to exert both a pro‐nociceptive and an anti‐nociceptive effect (because high‐estradiol/low‐progesterone states are associated with decreased pain sensitivity and increased brain μ‐opioid receptor binding compared with low‐estradiol states, and decreased endogenous opioid neurotransmission is associated with low estradiol), testosterone, a typically male sex hormone, seems to have a greater anti‐nociceptive potential (Bartley & Fillingim, 2013; Craft, 2007; Smith et al., 2006).

Second, we identified that, regardless of sex, older adults presented higher HPT and lower perception of neutral and cold thermal stimuli, in addition to wet stimuli (across all temperatures, i.e., warm, neutral and cold hygrosensation). The present data are in line with previous findings indicating that the physiological process of ageing causes both structural and functional modification of the skin. As age advances, the skin becomes less elastic, more dehydrated and less vascularized and innervated (Bowden & McNulty, 2013a, 2013b; Carmeli et al., 2003). These alterations can lead to changes in skin sensitivity to potentially harmful stimuli, which might, in turn, represent a risk factor for the development of pressure (Kottner et al., 2019; Mervis & Phillips, 2019). As previously proposed, HPT appears to increase with age owing to changes in the structure and function of nociceptor subtypes and transmission pathways. For example, myelinated type Aδ fibres have been shown to present a longer latency in elderly subjects compared with younger people (Bowden & McNulty, 2013a; Carmeli et al., 2003; Gibson et al., 1991; Larivière et al., 2007; Mervis & Phillips, 2019).

Third, we found a generalized decrease in the perception of wetness in the older compared with the younger group. This observation could potentially be explained by the ageing‐induced sensory deficit mediated by skin changes and altered tactile sensitivity (Typolt & Filingeri, 2020). Our group has previously considered that ageing‐induced dehydration caused by the thinning of the epidermis and dermis, and probably the resulting decrease in skin mechanical resistance, might, in turn, lead to such changes in wetness perception in older adults (Wildgoose et al., 2021). However, a limitation of the present study is that we did not measure skin hydration at the tested skin sites, making such considerations somewhat speculative. Accumulation of wetness at the skin interface caused by prolonged exposure to different body fluids contributes to superficial structural modifications of the skin, which can, in turn, reduce its mechanical strength to pressure and shearing forces (Gray et al., 2011). As such, sensitivity deficits associated with the perception of wetness build‐up at the skin could be considered a risk factor for the development of pressure ulcers. Elderly subjects, who can present other risk factors for pressure ulcers, appear to be subject to deficits in the perception of tactile and wet stimuli. This sensory deficit might therefore cause a lack of feedback response, which allows them to dry the part or change position by reducing the pressure on the affected area (Mervis & Phillips, 2019).

Fourth, our study provides new evidence on the body regional variation in mechanical, thermal and wetness perception across three skin sites that are at higher risk of developing pressure ulcers, yet typically little investigated in somatosensory research. Such literature has previously indicated variability of skin sensitivity, for example, to wetness between different skin sites, but usually these differences seem to occur in the presence of cold wet stimuli and not at warmer temperatures (Valenza et al., 2019). In the present study, differences between skin sites were found during both warm and cold stimuli. At the level of the sacrum and sternum, a different ability to perceive wetness at warm temperatures was highlighted. In particular, the sacrum seems to have a greater intensity of sensations to wetness at warmer temperatures compared with the sternum. In contrast, differences between the sacrum and the heel for intensity of sensation to wetness at cold temperatures were also found, because the sacrum seems to present a higher intensity of sensation to wetness at cold temperatures but not for warm and neutral wet stimuli. Likewise, the perception of mechanical pain was found to be different according to the investigated skin site, and although the heel was typically the least sensitive skin site, age was found to modulate PPT differently, in that older adults were characterized by lower thresholds at the heel and higher thresholds at the sacrum compared with young adults. These differences in the level of perception of the different sensory modalities between skin sites could help in prevention of the development of pressure ulcers by modifying the interventions to be implemented based on the affected skin site.

This study presents some limitations, including the fact that it was not possible simultaneously to evaluate the three extrinsic factors, namely mechanical, thermal and wetness perception. In real‐life conditions, thermal, mechanical and moisture factors are likely to interact to increase the risk of pressure ulcers. Future studies should therefore consider investigating perceptual differences in relationship to thermal and wet stimuli applied at different pressure levels. Furthermore, given that skin dehydration is a typical phenomenon occurring with advancing age and represents a key factor that seems to be involved in the loss of tactile/mechanical sensitivity, future studies might consider evaluating the level of local skin hydration at tested sites. Finally, the sample size could be expanded to be more accurate and to allow further subgroup analysis based on other individual factors. Despite these limitations, the present study provides some important new insights on the sensory capacity of the skin to perceive mechanical, thermal and wetness stimuli in skin sites that have been little studied, and it encourages future studies to translate this protocol to the clinic, to assess how prolonged pressure, the main risk factor for the development of pressure ulcers, can alter these investigated sensitivities.

Taken together, our findings suggest that both sex and ageing factors induce alterations in skin perception at skin sites at risk of pressure ulcers. In particular, age and sex can represent independent factors associated with different intensity of sensations, and such differences vary with the skin site being examined (mostly the heel and sacrum); as such, considering altered mechanical, thermal and wetness perceptions as risk factors for the development of pressure ulcers, male adults >50 years of age could be at more risk than younger individuals, particularly on the heel and sacrum.

## 5 CONCLUSION

Skin sensing has been suggested to represent a fundamental ability to reduce the risk of pressure ulcers. Quantification of sensory capacity offers the opportunity to evaluate mechanical, thermal and wetness perception on different skin sites and in different populations. The results from this study provide insights regarding skin perception in healthy subjects for specific skin sites where the risk of pressure ulcers could be influenced by intrinsic factors, such as age and sex. Male sex, age and skin site (mainly the heel and sacrum) are characterized by reduced intensities of sensations to mechanical, thermal and wetness stimuli. Quantitative sensory assessment of these areas could be applied in the clinical setting to identify those subjects with alterations skin sensation, who might be at a higher risk of developing pressure ulcers.

## AUTHOR CONTRIBUTIONS

Miriam Martini, Arianna Ortis, Davide Filingeri and Alex Buoite Stella conceived and designed the work. Miriam Martini, Arianna Ortis, Evelin Makuc and Manuela Deodato acquired the data. Miriam Martini, Peter R. Worsley and Alex Buoite Stella analysed and interpreted the data. Miriam Martini and Alex Buoite Stella drafted the manuscript, and all authors revised the manuscript critically for intellectual content. All authors approved the final version of the manuscript and agree to be accountable for all aspects of the work in ensuring that questions related to the accuracy or integrity of any part of the work are appropriately investigated and resolved. All persons designated as authors qualify for authorship, and all those who qualify for authorship are listed.

## CONFLICT OF INTEREST

None declared.

## Data Availability

Anonymized data can be requested upon reasonable request to the corresponding author.
